# An Improved HPLC-DAD Method for Quantitative Comparisons of Triterpenes in *Ganoderma lucidum* and Its Five Related Species Originating from Vietnam

**DOI:** 10.3390/molecules20011059

**Published:** 2015-01-09

**Authors:** Do Thi Ha, Le Thi Loan, Tran Manh Hung, Le Vu Ngoc Han, Nguyen Minh Khoi, Le Viet Dung, Byung Sun Min, Nguyen Phuong Dai Nguyen

**Affiliations:** 1 National Institute of Medicinal Materials (NIMM), 3B Quangtrung, Hoankiem, Hanoi 100000, Vietnam; E-Mails: vloandkh@gmail.com (L.T.L.); levungochan@gmail.com (L.V.N.H.); khoi_nguyenminh@yahoo.co.uk (N.M.K.); levietdung75imm@yahoo.com (L.V.D.); 2 Faculty of Chemistry, University of Science, Vietnam National University-HoChiMinh City, 227 Nguyen Van Cu, District 5, Ho Chi Minh City 227-01, Vietnam; 3 College of Pharmacy, Catholic University of Daegu, Gyeungbuk 712–702, Korea; E-Mail: bsmin@cu.ac.kr; 4 Department of Biology, University of Taynguyen, 567 Le Duan, Dac Lac City 630000, Vietnam; E-Mail: nguyendhtn@gmail.com

**Keywords:** *Ganoderma lucidum*, related linhzhi species, HPLC-DAD, triterpenes, quantitative analysis

## Abstract

An HPLC-DAD method for the quality control of wild and cultivated *Ganoderma lucidum* (Linhzhi) and related species samples was developed and validated. The quantitative determination of *G. lucidum* and its related species using 14 triterpene constituents, including nine ganoderma acids (compounds **4**–**12**), four alcohols (compounds **13**–**16**), and one sterol (ergosterol, **17**) were reported. The standard curves were linear over the concentration range of 7.5–180 µg/mL. The LOD and LOQ values for the analyses varied from 0.34 to 1.41 µg/mL and from 1.01 to 4.23 µg/mL, respectively. The percentage recovery of each reference compound was found to be from 97.09% to 100.79%, and the RSD (%) was less than 2.35%. The precision and accuracy ranged from 0.81%–3.20% and 95.38%–102.19% for intra-day, and from 0.43%–3.67% and 96.63%–103.09% for inter-day, respectively. The study disclosed in detail significant differences between the quantities of analyzed compounds in different samples. The total triterpenes in wild Linhzhi samples were significantly higher than in cultivated ones. The total constituent contents of the five related Linhzhi samples were considerably lower than that in the *G. lucidum* specimens, except for *G. australe* as its constituent content outweighed wild Linhzhi’s content by 4:1.

## 1. Introduction

*Ganoderma lucidum* (Leyss. Ex Fr.) Karst (Linhzhi) is an ancient traditional medicine that is highly valued in Asian countries as well as in the West for the treatment and prevention of many diseases such as neurasthenia, insomnia, anorexia, dizziness, chronic hepatitis, hypercholesterolemia, mushroom poisoning, coronary heart disease, hypertension, and carcinoma [1,2]. Linhzhi has been considered a tincture of life for thousands of years. Currently, a number of Linhzhi commercial products such as teas, capsules, tablets, raw herb, powders, and extracts are available and are used medicinally [1,2,3]. A number of bioactive components from *G. lucidum* have been identified, including polysaccharides, triterpenoids, lectins, steroids, and proteins, which have been used in the treatment and the prevention numerous of diseases [2]. Among those components, triterpenoids, including highly oxygenated lanostane derivatives and common fungal steroids derived from ergosterol, have received considerable attention owing to their well-known pharmacological activities such as anti-HIV-1 (ganoderiol F, ganodermanontriol, ganoderic acids A, B, H, and C1) [4], anticholesterol (ganoderic acids A, B, and C) [5], antinociceptive (ganoderic acids A, B, G, and C6) [6], hepatoprotective (ganodermanontriol, ergosterol) [7], antihistamine (ganoderic acids C2 and D) [8], and antitumor (ganoderic acids T, Me) [9] activities, as well as H/R-induced oxidative stress and inflammatory responses (ergosterol) [10]. Hence, triterpenoids could be considered the “marker compounds” for the chemical evaluation and the standardization of the bioactive components from *G. lucidum*. The chemical structures of the triterpenoids in *G. lucidum* are quite similar and difficult to isolate in sufficient amounts for the simultaneous analysis of multiple components. Therefore, the quality control and standardization of the isolation of the components of *G. lucidum* is a challenging task. However, high-performance liquid chromatography (HPLC) has been used successfully to overcome these difficulties [11,12,13,14].

Despite the valuable dietary and therapeutic benefits of Linhzhi, chemical investigations of the active components have been conducted mainly in China, Korea, Japan, and United States, but few experiments with the local *Ganoderma* genus (the family: Ganodermataceae) have been carried out [12,15,16]. In Vietnam, several studies have been reported regarding the taxonomy and distribution of Vietnamese Ganodermataceae [17,18]. Vietnam possesses 27 species of the *Ganoderma* genus [18]; however, the classification and distribution of the *Ganoderma* genus in Vietnam continues to be studied. In a 2013 report by Nguyen *et al.*, 43 species of the *Ganoderma* genus were identified in the Highlands of Vietnam [17]. Five of these have been successfully cultivated in Vietnam including *G. lucidum*, *G. applanatum*, *G. australe*, *G. colossum*, and *G. subresinosum*. However, the chemical compositions of the *G. lucidum* species collected in Vietnam have not been reported, and no data exist that compare the components of wild-harvested, cultivated, and other related Linhzhi species from Vietnam. In this study, a reverse-phase HPLC method was developed for the fingerprint analysis and simultaneous determination of 17 compounds including a new lanostane triterpene (butyl lucidenate E2 (**11**) [19], uracil (**1**), 5-dihydrobenzoic acid (**3**, gentisic acid) [20], 12 lanostane triterpene derivatives (compounds **4**–**10** and **12**–**16**), adenosine (**2**), and ergosterol (**17**). The developed method was successfully applied to the quantification of 14 triterpenoids in six wild and four cultivated *G. lucidum* samples, and five related *Ganoderma* species.

## 2. Results and Discussion

### 2.1. Optimization of Sample Preparation Condition

Several methods for the extraction of the fruiting bodies of *Ganoderma* species were surveyed including ultrasonication, refluxing, and maceration using methanol, but the ultrasonication method was the most effective, therefore we used this method to evaluate the effect of different solvents (100% methanol and 100% ethanol) on the amount of sample extracted. When 100% methanol was used, the content of the sample extracted was higher. To test the necessary time to accomplish the extraction, samples were prepared for 30, 60, 90, and 120 min. Since, the amount of the sample extracted after 90 min was same as the 120 min sample and higher than the 30 min sample, 90 min was selected as the optimal extraction time.

### 2.2. Selection of HPLC Conditions and Validation of the Developed Method

**Figure 1:**
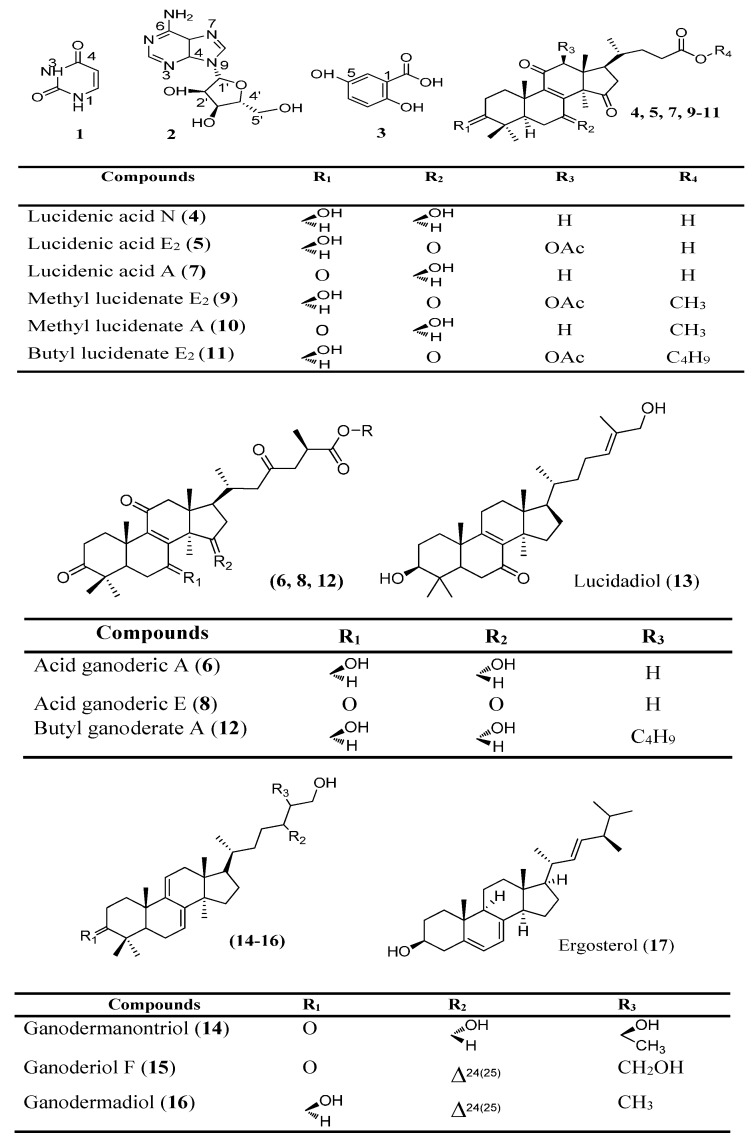
Chemical compounds **1**–**17** isolated from *G. lucidum*.

**Figure 2:**
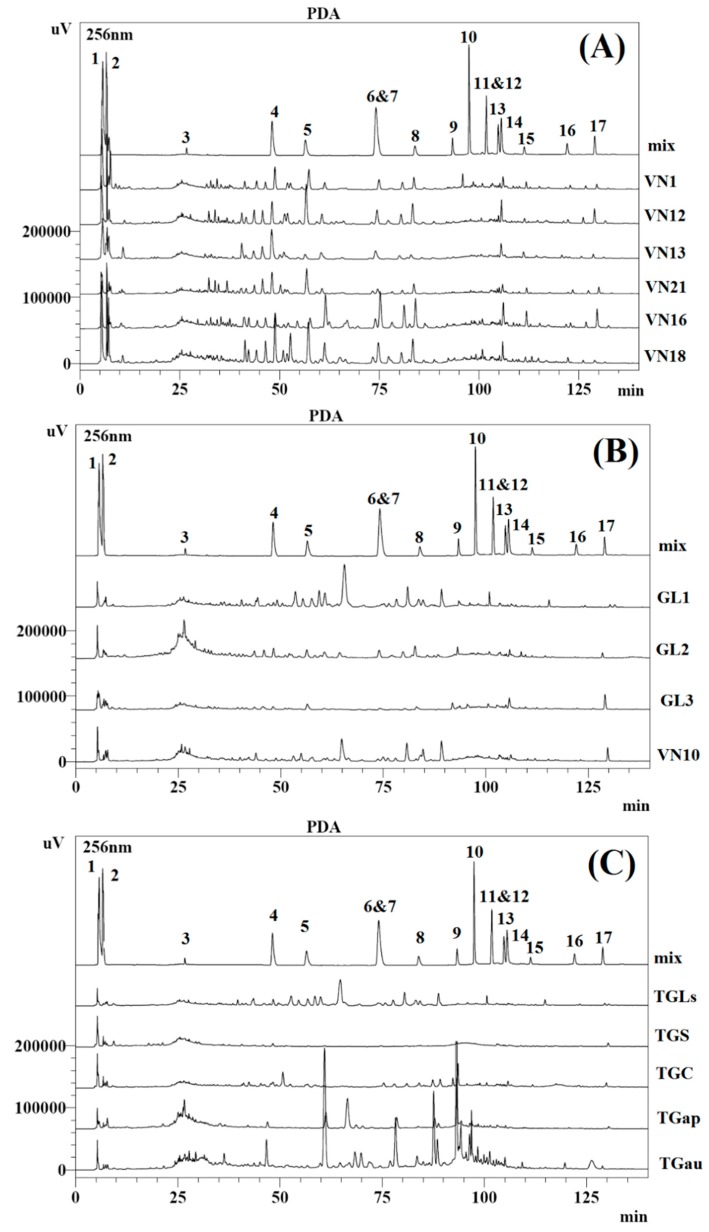
Chromatographic fingerprints of *Ganoderma* species. (**A**) the wild collected Linhzhi group (**Group A**); (**B**) the cultivated Linhzhi group (**Group B**); (**C**) the related Linhzhi species group (**Group C**). Compounds **1**–**17** were isolated from *Ganoderma lucidum* from the previous studies.

Although several HPLC methods have been reported for the determination of the constituents of Linhzhi samples [11,12,14,21,22], few constituents have been analyzed within the same study due to a lack of standard reference compounds. Our previous chemical investigation of *G. lucidum* from Vietnam resulted in the extraction and isolation of 17 compounds **1**–**17** (Figure 1). The chemical structures of the isolated compounds were identified using UV-Vis, IR, ^1^H- and ^13^C-NMR, and mass spectrometry as well as by the comparison of these spectroscopic data with those reported in literature as a new lanostane triterpene (butyl lucidenate E2 (**11**) [19], adenosine (**2**), and two compounds isolated from the first time in G*. lucidum*, namely uracil (**1**) and gentisic acid (**3**) [20]. In addition, 11 lanostane steroids were identified, including lucidenic acid N (**4**), lucidenic acid E2 (**5**), ganoderic acid A (**6**), lucidenic acid A (**7**), ganoderic acid E (**8**), methyl lucidenate E2 (**9**), methyl lucidenate A (**10**), and butyl ganoderate A (**12**) [23], lucidadiol (**13**), ganodermanontriol (**14**), ganoderiol F (**15**), ganodermadiol (**16**), and ergosterol (**17**) [19,20,24]. The purities of the compounds were greater than 95%, as estimated using an HPLC-DAD method. Most Linhzhi triterpenoids contain a conjugated skeleton, and their UV absorption peaks are concentrated at 210, 237, 243, 253, and 255 nm [11,16]. The analytes were divided into three groups including lanostane triterpenoid-type alcohols and acids, sterol, and others including gentisic acid and adenosine. Based on the maximum absorption of the compounds, the detection wavelengths were set at 256 nm for the acids and their derivatives and 243 nm for the others. The retention times of the compounds in the analyzed samples were distinguished by comparing with those of each reference compound, which are shown in Table 1. The sample preparation conditions for the extraction of the compounds in the *Ganoderma* species were optimized, which are described in Section 2.1. This study describes the results of the fingerprint analysis of the compounds **1**–**17** and the quality analysis of 14 of them (compounds **4**–**17**). The chromatographic fingerprints of the *Ganoderma* species are shown in Figure 2, which are divided into three groups including (**A**) the wild Linhzhi group, (**B**) the cultivated Linhzhi group, and (**C**) the related species group.

This HPLC-DAD method was validated for linearity, the limits of detection (LOD) and limits of quantitation (LOQ), recovery, and reproducibility. Each coefficient of correlation (*r*^2^) was >0.999, as determined by least square analysis, suggesting good linearity between the peak area ratio *versus* the compound concentration (Table 1). The LOD and LOQ were examined based on the lowest detectable peak in the chromatogram with a signal-to-noise (S/N) ratio of 3 and 10, respectively. Under our experimental conditions, we determined the LOD and LOQ for the 14 reference compounds in Table 2. The values obtained for both the LOD and LOQ in these analyses were low enough to detect traces of the compounds in the crude extract.

**Table 1:** Retention time reproducibilities, regression equations, correlation coefficients (*r*^2^), F-test, p (F), linearity ranges, LOD, and LOQ of the compounds **4**–**17** (*n* = 8).

Compounds	Retention Time Reproducibility (min, *n* = 8)	Regression Equation	*r*^2^	*F	*p (F)	Linearity Range (μg/mL)	LOD (μg/mL)	LOQ (μg/mL)
***Acid***								
Lucidenic acid N (**4**)	48.176 ± 0.110	*y* = 20041.5*x* − 11913.8	0.9999	12.67	0.00314	7.50–180.00	0.88	2.64
Lucidenic acid E2 (**5**)	56.463 ± 0.161	*y* = 10679.7*x* − 4195.8	0.9999	12.74	0.00308	7.50–180.00	0.82	2.45
Ganoderic acid A (**6**) & Lucidenic acid A (**7**)	74.075 ± 0.130	*y* = 19900.9x − 11310.7	0.9999	12.77	0.00305	7.50–180.00	0.34	1.01
Ganoderic acid E (**8**)	83.898 ± 0.240	*y* = 5790.0*x* − 4699.3	0.9999	12.60	0.00320	7.50–180.00	1.17	3.52
Methyl lucidenate E2 (**9**)	93.330 ± 0.083	*y* = 6087.3*x* − 5267.7	0.9999	12.59	0.00321	7.50–180.00	0.91	2.74
Methyl lucidenate A (**10**)	97.480 ± 0.080	*y* = 31602.7*x* − 12081.8	0.9999	12.74	0.00308	7.50–180.00	0.68	2.04
Butyl lucidenate E2 (**11**) & Butyl ganoderate A (**12**)	101.827 ± 0.116	*y* = 9737.0*x* − 12899.6	0.9999	12.65	0.00316	7.50–180.00	1.10	3.31
***Alcohol***								
Lucidadiol (**13**)	104.729 ± 0.115	*y* = 9573.6*x* − 3631.9	0.9999	12.74	0.00308	7.50–180.00	0.57	1.72
Ganodemanontriol (**14**)	105.589 ± 0.213	*y* = 34791.4*x* + 110902.3	0.9999	13.89	0.00226	7.50–180.00	1.39	4.18
Ganoderiol F (**15**)	111.230 ± 0.245	*y* = 7417.9*x* − 14493.5	0.9999	12.25	0.00353	7.50–180.00	1.41	4.23
Ganodermadiol (**16**)	121.772 ± 0.258	*y* = 11250.1*x* − 5594.5	0.9999	12.70	0.00311	7.50–180.00	1.37	4.12
***Other***								
Ergosterol (**17**)	128.915 ± 0.267	*y* = 18821.0*x* − 5702.9	0.9999	12.77	0.00306	7.50–180.00	0.94	2.82

* F-test and p (F) values were analysed by one-way analysis of variance (ANOVA using the Origin V7.5 software (Originlab, Northampton, MA, USA). Statistical significance was assumed at below the 0.05 probability level.

**Table 2:** Recovery rates of the compounds **4**–**17** from extracts of *G. lucidum*.

Compounds	Added (µg/mL)	Detected (µg/mL)	Recovery (%)	M ± SD (%)	RSD (%)
***Acid***					
Lucidenic acid N (**4**)	9.50	9.47	99.68	99.51 ± 1.48	1.49
	19.00	19.17	100.89		
	38.00	37.22	97.95		
Lucidenic acid E2 (**5**)	10.20	10.23	100.29	98.44 ± 1.61	1.64
	20.40	19.92	97.65		
	40.80	39.73	97.38		
Ganoderic acid A (**6**) & Lucidenic acid A (**7**)	10.40	10.21	98.17	98.71 ± 1.23	1.25
	20.80	20.35	97.84		
	41.60	41.65	100.12		
Ganoderic acid E (**8**)	10.80	10.54	97.59	99.20 ± 1.45	1.46
	21.60	21.69	100.42		
	43.20	43.02	99.58		
Methyl lucidenate E2 (**9**)	10.00	10.28	102.80	100.79 ± 1.74	1.73
	20.00	19.95	99.75		
	40.00	39.93	99.83		
Methyl lucidenate A (**10**)	10.10	9.82	97.23	98.39 ± 1.76	1.79
	20.20	19.70	97.52		
	40.40	40.57	100.42		
Butyl lucidenate E2 (**11**) and Butyl ganoderate A (**12**)	10.10	9.93	98.32	97.52 ± 1.95	2.00
	20.20	19.25	95.30		
	40.40	39.97	98.94		
***Alcohol***					
Lucidadiol (**13**)	10.50	10.12	96.38	97.17 ± 2.28	2.35
	21.00	20.03	95.38		
	42.00	41.89	99.74		
Ganodemanontriol (**14**)	10.20	10.12	99.22	99.32 ± 1.27	1.27
	20.40	20.53	100.64		
	40.80	40.03	98.11		
Ganoderiol F (**15**)	10.40	10.05	96.63	97.09 ± 1.12	1.15
	20.80	20.46	98.37		
	41.60	40.05	96.27		
Ganodermadiol (**16**)	10.00	9.94	99.40	99.45 ± 1.63	1.63
	20.00	20.22	101.10		
	40.00	39.14	97.85		
***Other***					
Ergosterol (**17**)	10.40	10.07	96.83	97.70 ± 2.21	2.26
	20.80	19.98	96.06		
	41.60	41.69	100.22		

For the recovery, each reference compound was spiked into 1 g of each *Ganoderma* species at three levels, as described in the Experimental Section. The spiked samples were assayed, and the recoveries of each reference compounds were found to be 97.09% to 100.79%, and the relative standard deviation (RSD) (%) was less than 2.31% (Table 2). The average recovery was represented by the formula: R (%) = [(amount from the sample spiked standard − amount from the sample)/amount from the spiked standard] × 100. Table 3 shows the intra-day and inter-day precision (%RSD) of this HPLC method. The precision and accuracy ranged from 0.81%–3.20% and 95.38%–102.19% for intra-day and from 0.40%–3.67% and 95.63%–103.09% for inter-day, respectively. The data demonstrate that the method was acceptable in terms of linearity, accuracy, and reproducibility.

### 2.3. Quantitative Comparison of Different Ganoderma Species

The amount of the chemical compounds within the samples was influenced by various factors such as the place of origin, type of study sample (cultivated or wild samples; different species of the same genus), and harvesting season. The variation of the lanostane triterpenoid alcohol or acid derivatives and ergosterol in the different *Ganoderma* species originating from the wild and cultivated collections from Vietnam was evaluated. The fingerprint analysis of the wild Linhzhi group (Figure 2A) showed a similarity across the chromatograms, and the 17 analytes were present in all the samples. Figure 2B,C show the differences among the wild Linhzhi group (A group), the cultivated Linhzhi group (B group), and the related Linhzhi species group (C group). In particular, the chromatograms of the five related Linhzhi species including *G. sp*, *G. applanatum*, *G. australe*, *G. colossum*, and *G. subresinosum* showed obvious differences. Compounds **1**–**3** were not distinctly separated using the developed method, so they were not quantified. This study focused on the simultaneous determination of the remaining 14 compounds **4**–**17** by using the developed HPLC-DAD method for the all samples (Groups A, B and C), which are summarized in Table 4, Table 5 and Table 6, respectively. Each sample was analyzed in triplicate to ensure the reproducibility of the quantitative results. The comparison of the 14 compounds in the Linhzhi samples (both wild and cultivated samples) showed that the number of acids and their derivatives in all the samples is significantly higher than the number of alcohols. However, there are differences between the wild and cultivated samples. While the total amount of the acids and alcohols in the wild samples vary from 2089.40 to 44,703.07 μg/g and 917.41 to 2498.68 μg/g, respectively, those from the cultivated samples fluctuate between 1003.83 and 1720.69 μg/g and 153.31 to 549.32 μg/g, respectively. Similarly, the total amount of the acids in *G. australe* outweighs that of the wild and cultivated Linhzhi samples and other related Linhzhi species (Table 4, Table 5 and Table 6,) with a total amount of 19,999.28 μg/g. In addition, the amount of the 2 compounds **11** and **12** in the analyzed samples was below LOQ except for TGau, which had 131.29 ± 1.31 µg/g.

**Table 3:** Intraday and interday repeatability of **4**–**17**.

Compounds	Nominal Conc. (µg/mL)	Inter-Day			Intra-Day		
		Observed Conc. (M ± SD), (μg/mL)	Accuracy (%)	Precision (%)	Observed Conc. (M ± SD), (μg/mL)	Accuracy (%)	Precision (%)
Lucidenic acid N **(4)**	30.25	30.02 ± 0.13	99.24	0.43	30.17 ± 0.42	99.74	1.39
	60.50	60.13 ± 2.10	99.39	3.49	61.02 ± 1.95	100.86	3.20
	121.00	120.04 ± 2.29	99.21	1.91	120.11 ± 2.25	99.26	1.87
Lucidenic acid E2 **(5)**	29.75	30.33 ± 0.12	101.95	0.40	30.12 ± 0.50	101.24	1.66
	59.50	60.17 ± 0.97	101.13	1.61	58.27 ± 0.79	97.93	1.36
	119.00	118.56 ± 1.96	99.63	1.65	118.39 ± 0.96	99.49	0.81
Ganoderic acid A **(6)** & Lucidenic acid A **(7)**	30.75	30.28 ± 1.11	98.47	3.67	30.10 ± 0.59	97.89	1.96
	61.50	60.00 ± 1.05	97.56	1.75	59.88 ± 0.88	97.37	1.47
	123.00	122.12 ± 1.27	99.28	1.04	121.16 ± 2.05	98.50	1.69
Ganoderic acid E **(8)**	29.75	29.59 ± 0.59	99.46	1.99	29.03 ± 0.76	97.58	2.62
	59.50	59.27 ± 1.12	99.61	1.89	58.36 ± 0.99	98.08	1.70
	119.00	118.33 ± 0.95	99.44	0.80	118.13±1.13	99.27	0.96
Methyl lucidenate E2 **(9)**	28.75	27.94 ± 0.84	97.18	3.01	29.13 ± 0.89	101.32	3.06
	57.50	58.24 ± 1.10	101.29	1.89	56.02 ± 0.93	97.43	1.66
	115.00	116.05 ± 1.27	100.91	1.09	114.12 ± 2.11	99.23	1.85
Methyl lucidenate A **(10)**	31.75	29.62 ± 0.66	93.29	2.23	30.59 ± 0.73	96.35	2.39
	63.50	62.21 ± 0.98	97.97	1.58	62.58 ± 1.06	98.55	1.69
	127.00	125.76 ± 0.74	99.02	0.59	126.29 ± 1.94	99.44	1.54
Butyl lucidenate E2 **(11)** and Butyl ganoderate A **(12)**	33.75	29.45 ± 0.58	87.26	1.97	32.19 ± 0.82	95.38	2.55
	67.50	66.54 ± 1.20	98.58	1.80	66.83 ± 1.02	99.01	1.53
	135.00	133.50 ± 1.31	98.89	0.98	134.20 ± 2.07	99.41	1.54
Lucidadiol **(13)**	32.50	30.08 ± 0.69	92.55	2.29	32.19 ± 0.83	99.05	2.58
	65.00	63.89 ± 1.01	98.29	1.58	63.97 ± 1.32	98.42	2.06
	130.00	128.95 ± 1.17	99.19	0.91	128.04 ± 1.95	98.49	1.52
Ganodemanontriol **(14)**	30.75	29.85 ± 0.58	97.07	1.94	29.83 ± 0.59	97.01	1.98
	61.50	61.59 ± 0.94	100.15	1.53	60.82 ± 0.83	98.89	1.36
	123.00	122.11 ± 1.27	99.28	1.04	122.39 ± 1.96	99.50	1.60
Ganoderiol F **(15)**	29.25	29.78 ± 0.55	101.81	1.85	29.89 ± 0.55	102.19	1.84
	58.50	58.02 ± 1.01	99.18	1.74	58.01 ± 1.03	99.16	1.78
	117.00	115.38 ± 1.78	98.62	1.54	116.12 ± 2.11	99.25	1.82
Ganodermadiol **(16)**	30.25	30.05 ± 0.73	99.34	2.43	30.11 ± 0.49	99.54	1.63
	60.50	60.02 ± 0.79	99.21	1.32	60.12 ± 1.37	99.37	2.28
	121.00	120.11 ± 1.02	99.26	0.85	120.37 ± 2.05	99.48	1.70
Ergosterol **(17)**	29.75	30.67 ± 0.63	103.09	2.05	30.17 ± 0.73	101.41	2.42
	59.50	58.03 ± 0.92	97.53	1.59	59.20 ± 1.52	99.50	2.57
	119.00	119.97 ± 1.12	100.82	0.93	118.79 ± 1.99	99.82	1.68

**Table 4:** Contents of **4**–**17** in the wild collected Linhzhi samples (**Group A**).

Compounds	Content (µg/g) ^a^					
	VN1	VN12	VN13	VN16	VN18	VN21
***Acid***						
Lucidenic acid N (**4**)	388.16 ±5.10	444.58 ± 3.84	845.46 ± 9.89	420.71 ± 2.40	257.80 ± 3.88	884.05 ± 7.45
Lucidenic acid E2 (**5**)	776.90 ± 9.83	1766.75 ± 24.01	319.47 ± 6.93	1151.08 ± 7.64	430.79 ± 7.04	1695.01 ± 16.32
Ganoderic acid A (**6**) & Lucidenic acid A (**7**)	228.88 ± 3.12	401.93 ± 2.37	349.93 ± 5.05	140.97 ± 1.71	891.05 ± 7.68	509.76 ± 7.05
Ganoderic acid E (**8**)	878.27 ± 7.75	1528.73 ± 12.88	574.54 ± 4.71	797.83 ± 18.76	2100.20 ± 13.11 ^b^	1614.25 ± 9.27
Methyl lucidenate E2 (**9**)	−	−	−	−	−	−
Methyl lucidenate A (**10**)	46.62 ± 1.47	−	−	−	−	−
Butyl lucidenate E2 (**11**) & Butyl ganoderate A (**12**)	−	−	−	−	−	−
**Total acid**	**2318.82 ± 6.24**	**4141.98 ± 22.15**	**2089.40 ± 24.29**	**2510.58 ± 29.77**	**3679.85 ± 21.66**	**4703.07 ± 38.72**
***Alcohol***						
Lucidadiol (**13**)	144.44 ± 2.32	212.81 ± 1.35	111.31 ± 1.00	128.84 ± 0.54	243.95 ± 6.43	219.25 ± 2.00
Ganodemanontriol (**14**)	168.35 ± 2.53	364.27 ± 3.33	232.15 ± 1.82	129.31 ± 0.95	394.10 ± 5.78	291.04 ± 2.44
Ganoderiol F (**15**)	697.42 ± 13.75	643.68 ± 11.49	795.69 ± 5.07	563.94 ± 2.86	1635.06 ± 13.63	689.01 ± 2.02
Ganodermadiol (**16**)	218.99 ± 4.14	159.87 ± 0.95	91.38 ± 1.03	95.33 ± 0.60	225.56 ± 0.49	348.52 ± 4.90
**Total alcol**	**1229.20 ± 21.16**	**1380.63 ± 14.30**	**1230.54 ± 6.38**	**917.41 ± 3.65**	**2498.68 ± 7.41**	**1547.83 ± 9.55**
***Other***						
**Ergosterol (17)**	**205.82 ± 1.72**	**700.74 ± 4.76**	**222.27 ± 4.93**	**325.85 ± 1.87**	**862.09 ± 12.71**	**160.74 ± 4.56**

(−): Lower than test limit and could not be quantified; ^a^ Data were expressed as mean ± SD of three experiments; ^b^ Out of linear range.

**Table 5:** Contents of **4**–**17** in the cultivated Linhzhi samples (**Group B**).

Compounds	Content (µg/g) ^a^			
	GL1	GL2	GL3	VN10
***Acid***				
Lucidenic acid N (**4**)	139.08 ± 1.82	−	79.58 ± 1.47	52.53 ± 1.06
Lucidenic acid E2 (**5**)	481.31 ± 4.07	420.91 ± 2.78	357.89 ± 2.90	258.06 ± 1.31
Ganoderic acid A (**6**) & Lucidenic acid A (**7**)	83.35 ± 2.40	232.55 ± 5.66	70.17 ± 0.66	76.19 ± 0.18
Ganoderic acid E (**8**)	730.00 ± 4.27	85.54 ± 0.89	207.77 ± 1.79	773.92 ± 4.75
Methyl lucidenate E2 (**9**)	286.94 ± 2.33	446.45 ± 8.63	288.41 ± 1.73	−
Methyl lucidenate A (**10**)	−	−	−	−
Butyl lucidenate E2 (**11**) & Butyl ganoderate A (**12**)	−	−	−	−
**Total acid**	**1720.69 ± 9.23**	**1185.46 ± 3.01**	**1003.83 ± 5.02**	**1160.36 ± 7.87**
***Alcohol***				
Lucidadiol (**13**)	69.15 ± 1.40	49.43 ± 0.69	109.00 ± 1.00	50.47 ± 1.59
Ganodemanontriol (**14**)	107.04 ± 3.32	208.34 ± 2.62	50.85 ± 0.80
Ganoderiol F (**15**)	86.16 ± 1.24	65.03 ± 0.71	153.75 ± 0.92	226.71 ± 1.29
Ganodermadiol (**16**)	59.59 ± 0.60	78.23 ± 0.64	37.07 ± 0.41
**Total alcol**	**155.31 ± 1.38**	**281.10 ± 4.06**	**549.32 ± 3.44**	**365.10 ± 0.93**
***Other***				
**Ergosterol** (**17**)	**135.14 ± 1.52**	**194.68 ± 3.74**	**795.96 ± 5.65**	**647.79 ± 10.62**

(−): Lower than test limit and could not be quantified; ^a^ Data were expressed as mean ± SD of three experiments.

**Table 6:** Contents of 4–17 in the related Linzhi samples (Group C).

Compounds	Content (µg/g) ^a^				
	TGLs	TGap	TGau	TGc	TGs
***Acid***					
Lucidenic acid N (**4**)	144.18 ± 1.15	−	63.13 ± 1.45	207.73 ± 2.05	57.50 ± 0.65
Lucidenic acid E2 (**5**)	413.76 ± 2.04	−	121.65 ± 4.50	201.92 ± 2.45	−
Ganoderic acid A (**6**) & Lucidenic acid A (**7**)	89.18 ± 0.97	−	78.09 ± 2.97	118.24 ± 3.09	−
Ganoderic acid E (**8**)	475.23 ± 3.28	114.89 ± 1.89	1213.14 ± 15.26	337.62 ± 4.91	51.10 ± 1.30
Methyl lucidenate E2 (**9**)	−	2499.52 ± 17.65	18,247.10 ± 48.40 ^b^	1023.84 ± 28.71	−
Methyl lucidenate A (**10**)	−	−	144.89 ± 3.34	−	−
Butyl lucidenate E2 (**11**) & Butyl ganoderate A (**12**)	−	−	65.65 ± 0.65	−	−
**Total acid**	**1119.26 ± 4.48**	**2613.80 ± 17.08**	**19,999.28 ± 41.66**	**1889.35 ± 20.91**	**108.60 ± 1.07**
***Alcohol***					
Lucidadiol (**13**)	54.04 ± 1.32	70.36 ± 0.06	98.44 ± 1.19	46.08 ± 0.78	−
Ganodemanontriol (**14**)	−	72.99 ± 1.88	−
Ganoderiol F (**15**)	84.61 ± 0.85	−	126.42 ± 1.32	272.73 ± 3.49	56.58 ± 0.70
Ganodermadiol (**16**)	−	−	47.55 ± 0.71
**Total alcol**	**138.65 ± 1.48**	**70.36 ± 0.06**	**224.86 ± 1.23**	**439.35 ± 5.22**	**56.58 ± 0.70**
***Other***					
**Ergosterol** (**17**)	**116.32 ± 1.59**	**112.71 ± 0.52**	**148.30 ± 2.63**	**221.45 ± 2.49**	**158.35 ± 1.92**

(−): Lower than test limit and could not be quantified; ^a^ Data were expressed as mean ± SD of three experiments; ^b^ Out of linear range.

**Figure 3:**
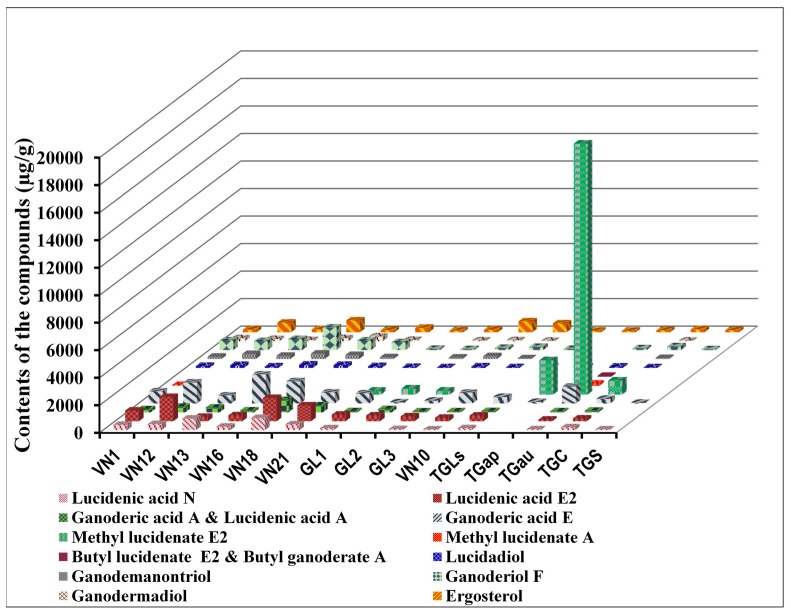
Content of **4**–**17** in various Linhzhi samples belong to groups A, B and C.

As shown in Table 4, Table 5, Table 6 and Figure 3, the total amount of all the compounds in the wild samples was appreciably higher than in the cultivated ones. For example, the amount of compound **4** (lucidenic acid N) in the wild *G. lucidum* sample varied from 257.80–845.46 μg/g; however, this compound was not observed in GL2, and in the other cultivated samples (Group C) it fluctuated between 52.53–139.08 μg/g. Another good example is lucidenic acid E2 (**5**), which was found in the wild *G. lucidum* samples in a range from 319.47 to 1,766.75 μg/g in comparison with the cultivated *G. lucidum* samples in a range from 258.06 to 481.31 μg/g. In addition, the wild samples contained significantly more ganoderiol F (**15**) than the cultivated samples, which varied between 563.94 μg/g and 1,635.06 μg/g and between 65.03 μg/g and 226.71 μg/g, respectively. Interestingly, a different trend was observed for methyl lucidenate E2 (**9**), which was under the LOQ in the wild samples but was found in GL1, GL2, and GL3 in the range between 286.94 and 446.95 μg/g. On the whole, the samples from Bac Giang (VN16 and VN18) seemed to have a higher amount of the constituents than the specimens from Quang Nam (VN1, VN12, and VN13). The total amount of constituents in the related Linhzhi samples (Group C) including in TGau, TGLs, TGap, TGc, and TGs was considerably lower than the amount in the Linhzhi samples of Groups A and B. The amount of constituents in TGau outweighed that of the others, as it was about 4 times as high as that of the wild Linhzhi samples (VN12).

The proportion of constituents in the other species is different from *G. lucidum*. More specifically, the proportion of lucidenic acid E2 (**5**), which is one of the major compounds in *G. lucidum*, is low in TGap and TGS and is not found in TGau. Similarly, while almost *G. lucidum* samples contain a considerable amount of ganodemanontriol, it was only seen in trace amounts in the TGLs, TGap, TGau and TGs samples. In contrast, the amount of methyl lucidenate E2 (**9**), which is not observed in *G. lucidum*, is the major compound in the TGap, TGau, and TGc samples. It is noteworthy that the amount of the constituents in TGs was substantially smaller than others, and 50 percent was ergosterol.

### 2.4. Discussion

To date, several previous studies have reported using HPLC analytical methods for the analysis of *Ganoderma lucidum* and its related products*.* For example, Zhao *et al.* used HPLC for the determination of 9 triterpenes and sterols for the quality evaluation of *G. lucidum* [25]. In a study from Wang *et al.*, an RP-HPLC method was developed for the determination of six ganoderic acids [22]. In 2004, Gao and coworkers reported the quantitative determination of 19 triterpene constituents, including six ganoderma alcohols and 13 ganoderic acids [11]. These studies and others focused only on the ganoderic acids and their derivatives [12,26]. Nucleosides, nucleobases, and polysaccharides were used for the qualitative and quantitative analyses of *Ganoderma* spp [27]. However, these studies are insufficient for a comprehensive evaluation of *G. lucidum,* and there is little data that compare *G. lucidum* from different origins or compare *G. lucidum* and its related species*.*

In this paper, we developed and optimized an HPLC-DAD method that allows for the specific identification of many terpenes. Fourteen triterpenes, including nine ganodermic acids **4**–**12**, four alcohols **13**–**16**, and one sterol (ergosterol, **17**), were used for the quantitative determination of *G. lucidum* and its related species. Eight of the nine ganoderma acids (all but ganoderic acid A) had never been analyzed before. Two new lanostane triterpenes **11** and **12**, which recently were discovered by our group and Lee Iksoo *et al.* [24], were used in the fingerprint analysis and quantitative determination for the first time [19,23]. Alcohols **13** and **16**, which had never been examined quantitatively in previous studies, were used to evaluate *G. lucidum* and its related species chemically using HPLC-DAD. Two *Ganoderma* species investigated in this study were studied quantitatively using HPLC for the first time, except for *G. applanatum* [28]. However, in a study by Liu *et al.*, *G. applanatum* was evaluated by using five ganoderic acids. Moreover, two compounds, uracil and gentisic acid, which were found in *G. lucidum* for the first time, were confirmed using the HPLC fingerprint technique.

In comparison with the results of previous studies, this study showed both similarities and differences. In a study by Gao *et al.* [11], the amount of ganondermanontriol (**14**) in Japanese Linhzhi ranged widely from 19.2 to 235.3 μg/g. In our study, there was a wide variation of compound **14** in the wild, cultivated, and related Linhzhi samples ranging from 129.31 to 394.10 μg/g, 50.85 to 208.34 μg/g, and 72.99 ± 1.88 μg/g (related Linhzhi species, TGc), respectively. Therefore, the amount of compound **14** in the Japanese samples was similar to the Vietnamese cultivated samples but was lower than that found in the Vietnamese wild Linhzhi*.* These results indicate that the amount of compound **14** may not depend on geographic factors but instead is affected by the cultivation conditions. Gao’s study showed that the contents of ganoderiol F (**15**) ranged from 18.9 to 156.5 μg/g [11]. However, the Vietnamese wild samples contained 563.94–1635.06 μg/g of **15**, and the Vietnamese cultivated samples contained 65.03–226.71 μg/g of **15**. Both the wild and cultivated Linhzhi samples from Vietnam contain more compound **15** than the Linhzhi from Japan. Similar to the results found with compound **14**, these results indicate that amount of **15** is not only affected by geographic factors but also by cultivation conditions. In a study from the Yuan group, the content of ergosterol (**17**) from sporoderm-broken germinating spores of Linhzhi varied from 32 μg/g to 1202 μg/g in the cultivated Linhzhi from China [21], corresponding with the results from this study, as the content of **17** in the cultivated Linhzhi ranged between 135.14 μg/g to 795.96 μg/g. With regard to methyl lucidenate E2 (**9**), there was a huge difference in the amounts found among the wild and cultivated Linhzhi and its related species. While a quantitative determination of **9** in the wild species could not be made, its content was above 288 μg/g in the cultivated species. Interestingly, compound **9** was the major compound in related species of Linhzhi as its content was at least 1023.84 μg/g across the species and was as high as 2499.52 μg/g in *G. australe*.

## 3. Experimental Section

### 3.1. Ganoderma Species

The wildly collected samples (Group A) (*Ganoderma lucidum*) were VN1, VN12; VN13 originating from Tienphuoc, Quangnam Province; VN16 and VN18 originating from Bacgiang Province; and VN21 originating from Trami, Quangnam Province. The cultivated samples (Group B) (*Ganoderma lucidum*) were VN10 from Vietnam Academy of Agricultural Sciences, GL1-GL3 from Linh chi Vina company (Ho Chi Minh, Vietnam). The other species of *Ganoderma* genus (Group C) including *G. applanatum* (TGap), *G. colossum* (TGc), *G. subresinosum* (TGs), *Ganoderma sp* (TGls), and *G. australe* (TGau) were gifts from Linh chi Vina Company. The samples were botanically identified by Msc. Co Duc Trong, Linh chi Vina Company, where the voucher specimens were deposited. The other specimens of the test samples have been verified by KiHwan Bae from College of Pharmacy, Chungnam National University, Korea and deposited in the Department of Phytochemistry, NIMM, Vietnam. All dried samples were ground and transferred to the laboratory for preparation of the plant extracts.

### 3.2. Standard Compounds

The compounds **1**–**17** were isolated from the chloroform- and water-soluble fractions of the methanol extracts from fruiting bodies of *Ganoderma lucidum*, which were published previously by our group including a new lanostane triterpene butyl lucidenate E2 (**11**) and adenosine (**2**) and two compounds isolated from the first time in G*. lucidum*, namely uracil (**2**) and gentisic acid (**3**), 12 lanostane steroids including lucidenic acid N (**4**), lucidenic acid E2 (**5**), ganoderic acid A (**6**), lucidenic acid A (**7**), ganoderic acid E (**8**), methyl lucidenate E2 (**9**), methyl lucidenate A (**10**), butyl ganoderate A (**12**), lucidadiol (**13**), ganodermanontriol (**14**), ganoderiol F (**15**), ganodermadiol (**16**), and ergosterol (**17**) [19,20,24] (Figure 1). All chemical standards were isolated from *G. lucidum*, theirs purities were checked by the HPLC-DAD method and the chemical structures were elucidated by ^1^H-NMR, IR, UV/VIS spectroscopic analysis and MS spectral data.

### 3.3. Chemicals and Reagents

Acetonitrile and methanol (MeOH) of analytical HPLC grade was purchased from Merck (Darmstadt, Germany). Phosphoric acid of analytical reagent grade was obtained from Sigma-Aldrich (St Louis, MO, USA). The other organic solvents and other chemical reagents were of analytical reagent grade.

### 3.4. Reference Compound Preparation

To determine the content of fourteen markers (compounds **4**–**17**) of Linhzhi and related Linhzhi samples, the dried powders were used for extraction. The same amounts (about 1 g) of pulverized fruiting bodies were weighed and sieved through 50 mesh and then placed into a volumetric flask, methanol (10 mL) was added, the weight was accurately measured and the samples were ultrasonically extracted for 90 min at 50 °C. The solution was cooled, weighed again, and made up the loss in weight with methanol. The solution was filtered through 0.45 µm membrane filter prior to HPLC analysis.

### 3.5. HPLC

Analytical HPLC was carried out on a LC 20A system (Shimadzu, city, Japan) consisting of a LC-20AD quaternary gradient pump, an autosampler, and a SPD-M20A diode array detector, connected to a LC solution singer ver. 1.25 software. A Zorbax XDB C_18_ (4.6 × 250 mm, 5 µm, Agilent Technologies, Inc., Santa Clara, California, CA, USA) was used. A binary gradient solution system consisted of 0.1% phosphoric acid in water (A) and acetonitrile (B) and separation was achieved using the following gradient program: 0 min, 4% B; 10 min, 11% B; 15 min, 30% B; 60 min, 45% B; 90 min, 85% B; 110 min, 100 B%; 130–140 min, 100% B; and finally, reconditioning the column with 4% B isocratic for 10 min. The flow rate was 0.5 mL/min, the system operated at 40 °C and the detection wavelengths were set at 243 and 256 nm for ganoderma alcohols and acids, respectively.

### 3.6. Method Validation

Every standard compound was accurately weighed and dissolved in 100% MeOH to prepare a stock solution of 1.0 mg/mL concentration. Working standard solutions of ganoderma alcohols and acids were prepared by repeated dilution to give eight respective concentrations with methanol (7.5–180 µg/mL). Eight concentrations of 14 analyses were injected in triplicate, and then the calibration curves were constructed by plotting the peak areas *versus* the concentrations of each analysis. The linearity was demonstrated by a correlation coefficient (r2) greater than 0.999. The limit of detection (LOD) and the limit of quantification (LOQ) were determined based on signal-to noise ratios (S/N) of 3:1 and 10:1, respectively. Intra- and inter-day variations were chosen to determine the precisions of the developed method. The relative standard deviation (RSD) was taken as a measure of precision. Intra- and inter-day repeatability was determined on five times within one day and five separate days, respectively. The recovery tests were prepared by mixing a powdered sample (1 g) with three concentration levels (25%, 50%, and 100%) of each compound. The mixture was then extracted by following the section of preparation of sample solution for HPLC analysis. The extract solutions were filtered through a 0.45 µM membrane. The HPLC-DAD analysis experiments were performed in triplicate for each control level. Precision were determined by multiple analysis (*n* = 5) of quality control samples. All samples were then subjected to HPLC analysis to calculate the recovery rates.

### 3.7. Statistical Analysis

The data were analyzed using the unpaired Student’s *t*-test between the control and compounds. Data compiled from three independent experiments and values are expressed as mean ±SD.

## 4. Conclusions

This is the first time a HPLC-DAD method for quantitative analysis of constituents in *Ganoderma lucidum* and its related species batches originating from Vietnam was established. In the present work, we have reported for the first time the presence of lanostane triterpenes, ergosterol, uracil, adenosine, and gentisic acids in the Vietnamese *G. lucidum* and its related species. Especially, two new lanostanes, butyl lucidenate E2 and butyl ganoderate A, were reported for the first time in *G. lucidum* originating from Vietnam and its four related species using a HPLC-DAD method. In addition, the highest content of methyl lucidenate E2 was found in *G. australe*, *G. applatatum*, and *G. colossum*, respectively. In the present study the profile of the 17 compounds differed significantly in the all analyzed samples. It can be also concluded that the geographical distributions, growth conditions, and substrates might be the key to differences in producing chemical compositions. This present work suggested an accurate and sufficient method for quantitative evaluation, which is suitable for quality evaluation of *Ganoderma* products.

## Supplementary Materials

Supplementary materials can be accessed at: http://www.mdpi.com/1420-3049/20/01/1059/s1.

## References

[1] Long S.C., Birmingham J.M. (1992). Medicinal benefits of the mushroom *Ganoderma*. Adv. Appl. Microbiol..

[2] Boh B., Berovic M., Zhang J., Zhi-Bin L. (2007). *Ganoderma lucidum* and its pharmaceutically active compounds. Biotech. Ann. Rev..

[3] Kim H.W., Kim B.K. (1999). Biomedicinal triterpenoids of *Ganoderma lucidum* (Curt.: Fr.) P. Karst (aphyllophoromycetidae). Intern. J. Med. Mushrooms.

[4] El-Mekkawy S., Meselhy M.R., Nakamura N., Tezuka Y., Hattori M., Kakiuchi N., Shimotohno K., Kawahata T., Otake T. (1998). Anti-HIV-1 and anti-HIV-1-protease substances from *Ganoderma lucidum*. Phytochemistry.

[5] Kodora Y., Shimizu M., Sonoda Y., Sato Y. (1990). Ganoderic acid and its derivatives as cholesterol synthesis inhibitors. Chem. Pharm. Bull..

[6] Koyama K., Akiba M., Imaizumi T., Kinoshita K., Takahashi K., Suzuki A., Yano S., Horie S., Watanabe K. (2002). Antinociceptive constituents of *Auricularia polytricha*. Planta Med..

[7] Ha D.T., Oh J., Khoi N.M., Dao T.T., Dung L.V., Do T.N.Q., Lee S.M., Jang T.S., Jeong G.S., Na M. (2013). *In vitro* and *in vivo* hepatoprotective effect of ganodermanontriol against *t*-BHP-induced oxidative stress. J. Ethnopharmacol..

[8] Andoh T., Zhang Q., Yamamoto T., Tayama M., Hattori M., Tanaka K., Kuraishi Y. (2010). Inhibitory effects of the methanol extract of *Ganoderma lucidum* on mosquito allergy-induced itch-associated responses in mice. J. Pharmacol. Sci..

[9] Lindequist U., Niedermeyer T.H., Jülich W.D. (2005). The pharmacological potential of mushrooms. eCAM.

[10] Ma J.-Q., Liu C.-M., Qin Z.-H., Jiang J.-H., Sun Y.-Z. (2011). *Ganoderma applanatum* terpenes protect mouse liver against benzo(α)pyren-induced oxidative stress and inflammation. Environ. Toxicol. Pharmacol..

[11] Gao J.-J., Nakamura N., Min B.-S., Hirakawa A., Zuo F., Hattori M. (2004). Quantitative determination of bitter principles in specimens of *Ganoderma lucidum* using high-performance liquid chromatography and its application to the evaluation of ganoderma products. Chem. Pharm. Bull..

[12] Keypour S., Rafati H., Riahi H., Mirzajani F., Fata Moradali M. (2010). Qualitative analysis of ganoderic acids in *Ganoderma lucidum* from iran and china by rp-hplc and electrospray ionisation-mass spectrometry (ESI-MS). Food Chem..

[13] Ruan W., Popovich D.G. (2012). *Ganoderma lucidum* triterpenoid extract induces apoptosis in human colon carcinoma cells (Caco-2). Biomed. Prev. Nutr..

[14] Chen Y., Yan Y., Xie M.-Y., Nie S.-P., Liu W., Gong X.-F., Wang Y.-X. (2008). Development of a chromatographic fingerprint for the chloroform extracts of *Ganoderma lucidum* by hplc and LC–MS. J. Pharm. Biomed. Anal..

[15] Joseph S., Sabulal B., George V., Smina T.P., Janardhanan K.K. (2009). Antioxidative and anti-inflammatory activities of the chloroform extract of *Ganoderma lucidum* found in south india. Sci. Pharm..

[16] Salteralli R., Ceccaroli P., Lotti M., Zambonelli A., Buffalini M., Casadei L. (2009). Biochemical characterization and antioxidant activity of mycelium of *Ganoderma lucidum* from central italy. Food Chem..

[17] Nguyen P.D.N., Do H.T., Le B.D. (2013). Characteristics of ecological factors and their distribution of Ganodermataceae Donk. in highlands of vietnam. J. Biol..

[18] Dam N., Nguyen G.C., Nguyen B., Trinh T.K. (1997). *Ganoderma* species in vietnam. Hanoi-J. Med. Mat..

[19] Tung N.T., Cuong T.D., Hung T.M., Lee J.H., Woo M.H., Choi J.S., Kim J., Ryu S.H., Min B.S. (2013). Inhibitory effect on no production of triterpenes from the fruiting bodies of *Ganoderma lucidum*. Bioorg. Med. Chem. Lett..

[20] Ha D.T., Thu N.T., Khoi N.M. (2014). Derivatives of nucleic and benzoic acids isolated from *Ganoderma lucidum* collected in quangnam-danang, vietnam. Hanoi-J. Med. Mat..

[21] Yuan J.-P., Wang J.-H., Liu X., Kuang H.-C., Huang X.-N. (2006). Determination of ergosterol in ganoderma spore lipid from the germinating spores of *Ganoderma lucidum* by high-performance liquid chromatography. J. Agric. Food Chem..

[22] Wang X.M., Yang M., Guan S.H., Liu R.X., Xia J.M., Bi K.S., Guo D.A. (2006). Quantitative determination of six major triterpenoids in *Ganoderma lucidum* and related species by high performance liquid chromatography. J. Pharm. Biomed. Anal..

[23] Lee I., Seo J., Kim J., Kim H., Youn U., Lee J., Jung H., Na M., Hattori M., Min B. (2010). Lanostane triterpenes from the fruiting bodies of *Ganoderma lucidum* and their inhibitory effects on adipocyte differentiation in 3T3-L1 cells. J. Nat. Prod..

[24] Ha D.T., Thuy P.T., Khoi N.M. (2013). Ergostane and lanostane steroids from fruiting body of *Ganoderma lucidum* collected in quangnam, danang. Hanoi-J. Med. Mat..

[25] Zhao J., Zhang X.Q., Li S.P., Yang F.Q., Wang Y.T., Ye W.C. (2006). Quality evaluation of *Ganoderma* through simultaneous determination of nine triterpenes and sterols using pressurized liquid extraction and high performance liquid chromatography. J. Sep. Sci..

[26] Lu J., Qin J.-Z., Chen P., Chen X., Zhang Y.-Z., Zhao S.-J. (2012). Quality difference study of six varieties of *Ganoderma lucidum* with different origins. Front. Pharmacol..

[27] Gao J.L., Leung K.S.Y., Wang Y.T., Lai C.M., Li S.P., Hu L.F., Lu G.H., Jiang Z.H., Yu Z.L. (2007). Qualitative and quantitative analyses of nucleosides and nucleobases in *Ganoderma* spp. By HPLC-DAD-MS. J. Pharm. Biomed. Anal..

[28] Liu Y., Liu Q., Qiu F., Di X. (2011). Sensitive and selective liquid chromatography-tandem mass spectrometry method for the determination of five ganoderic acids in *Ganoderma lucidum* and its related species. J. Pharm. Biomed. Anal..

