# Supplementary figures and images for: An Improved HPLC-DAD Method for Quantitative Comparisons of Triterpenes in Ganoderma lucidum and Its Five Related Species Originating from Vietnam
Source: Molecules. 2015 Jan 9;20(1):1059–77. doi: 10.3390/molecules20011059 (PMC6272446; doi:10.3390/molecules20011059)

## Supplementary Materials

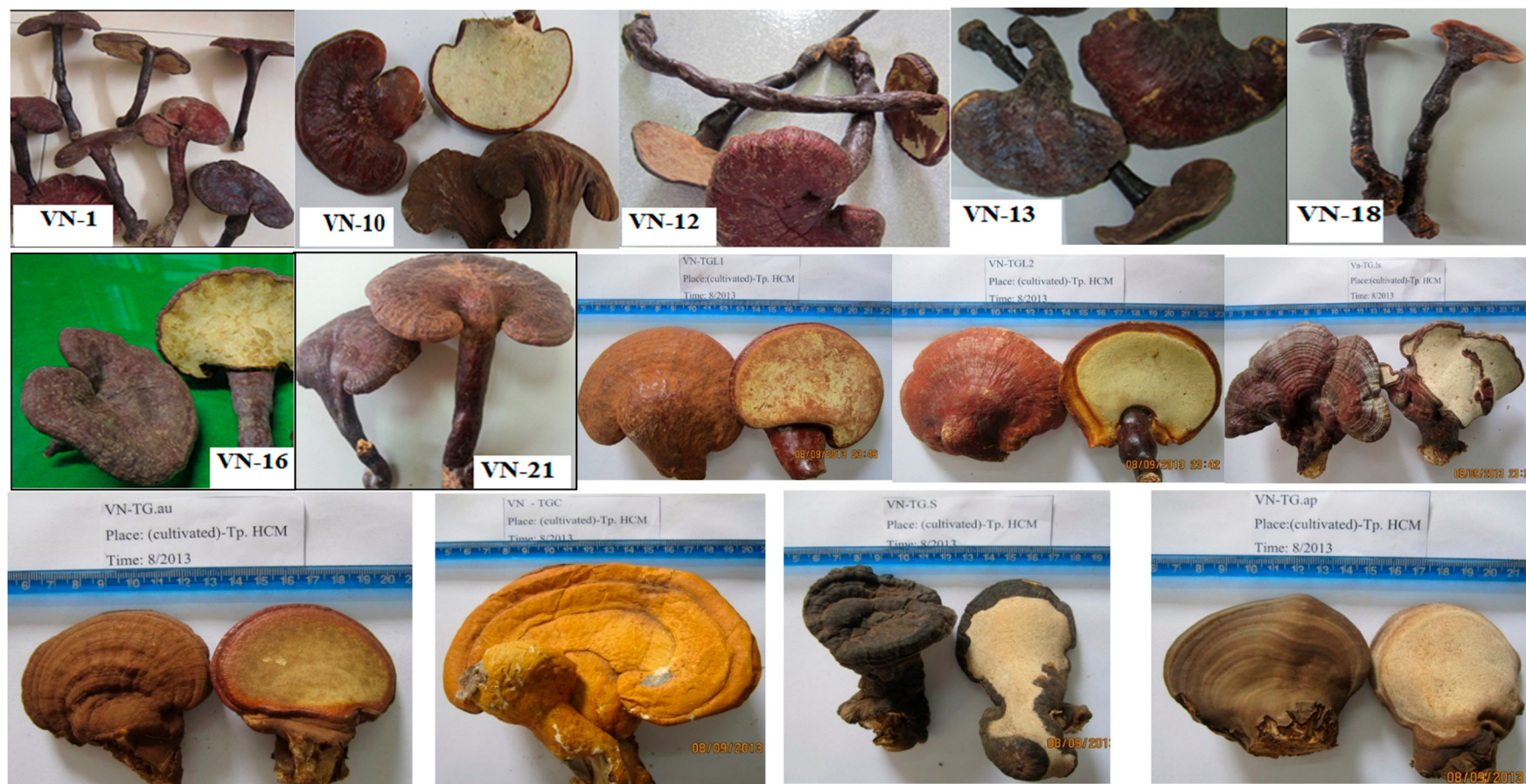

**Figure S1.** Pictures of Linzhzi and some related Linzhzi species.

Supplement: Supplementary file 1 [file molecules-20-01059-s001.pdf]
